# Algae and Microalgae and Their Bioactive Molecules for Human Health

**DOI:** 10.3390/molecules26041185

**Published:** 2021-02-23

**Authors:** Benoît Chénais

**Affiliations:** BiOSSE (Biology of Organisms: Stress, Health, Environment), UFR Sciences et Techniques, Le Mans Université, CEDEX 9, F-72085 Le Mans, France; bchenais@univ-lemans.fr

The algae and microalgae are an extremely diverse group of organisms that contain many bioactive molecules, including pigments, polyunsaturated fatty acids, polysaccharides, polyphenol, etc. Algae and microalgae and their bioactive molecules have a variety of health benefits such as antioxidant, anti-inflammatory, anticancer or antiobesity effects. However, due to the extraordinarily rich variety of species and molecules, one could be sure that new effects and/or new molecules are yet to be discovered. Thus, the objective of this special issue entitled “Algae and Microalgae and Their Bioactive Molecules for Human Health” is to present some recent advances in the description and understanding of the action of algal molecules or the effect of whole organism consumption on human health. It is also an opportunity to present new molecular and/or cellular mechanisms of action of bioactive molecules and new pathologies, such as nonalcoholic fatty liver disease (NAFLD) or viral infection, which could benefit from treatment with algae or microalgae. In addition, reports on the isolation and characterization of new bioactive compounds from algae and microalgae will also be of interest to further increase the already large chemodiversity. Altogether, this makes algae and microalgae great candidates for functional food and or preventive drugs. The use of microalgae as a bioengineering factory is also an avenue to be explored.

In the present Special Issue, the biodiversity and chemodiversity of macroalgae species was explored from brown seaweeds from the Russian coast [1] to chlorophyta from the Philippines [2] passing through other diverse species [3,4,5,6]. The study by Aminina et al. [1] investigated nine species of Laminariales and four species of Fucales of the Pacific coast of Russia for their composition in polyphenolic compounds with antioxidant activity. The phenolic content of algal extracts was dependent of the species. It was up to 3.5-fold higher in the ethanol extract than in the water extract for *Undaria pinnatifida*, *Arthrothamnus bifidus*, *Thalassiophyllum clathrus* and *Agarum turneri*. By contrast, it was around twofold higher in the water extract for *Sargassum pallidum* and *Kjellmaniella crassifolia*. The highest antioxidant activity was found for *Agarum turneri* ethanol extracts, which is also the species with the highest content of phlorotannin. Interestingly, the authors highlighted the presence of major phenols in the extract of *Thalassiophyllum clathrus*, such as phenolic acid (gallic acid), hydroxycinnamic acids (caffeic acid, chlorogenic acid, coumaric acid) and flavonols (kaempferol, quercetin).

The paper by Magdugo et al. [2] is focused on two poorly studied chlorophyta species, namely *Caulerpa racemosa* and *Ulva fasciata* from the Philippines. This research work highlighted the nutritional value of both algae, which contain high amount of proteins (i.e., 8.8–19.9% for *C. racemosa* and 8.0–11.1% for *U. fasciata*) with a portion of essential amino acids around 42–45% comparable to FAO/WHO requirements. However, the main essential amino acids were different in the two algae; leucine, valine, isoleucine, and lysine being the most abundant in *C. racemosa*, while leucine, valine, lysine, and phenylalanine dominate in *U. fasciata*. Concerning fatty acids, the two species are divergent since monounsaturated fatty acids and polyunsaturated fatty acids are dominant in *C. racemosa* (56.2%), while saturated fatty acids (72.1%) are prevailing in *U. fasciata*. Nevertheless, both species display a high ratio of C18/C20 polyunsaturated fatty acid. The main difference between these two species was the total pigment content, which was found 20-fold higher in *C. racemosa* (140.84 mg/g dw) compared to *U. fasciata* (7.54 mg/g dw). Mineral contents for both seaweeds were within levels considered safe for functional foods and the nutritional characteristics of *C. racemosa* make it interesting for human consumption and functional food ingredient. In addition, the hot water extract from *C. racemosa* showed in vitro antiherpetic activity without cytotoxicity.

Six other species of marine edible macroalgae have been investigated by Lopes et al. [3] for their content in lipids, especially omega-3 polyunsaturated fatty acids, and subsequent effects on various health-linked parameters. Lipid extracts from two green macroalgae, *Ulva rigida* and *Codium tomentosum*, three red seaweeds *Gracilaria gracilis*, *Palmaria palmata* and *Porphyra dioica*, and the brown algae *Fucus vesiculosus*, were obtained from a land-based integrated multitrophic aquaculture system and analyzed for their lipid quality indices based on their fatty acid profiles and their bioactivities. Results highlighted the species specificity of the health benefits. *U. rigida* displayed the lowest atherogenicity and thrombogenicity indices. The lipid extracts from *Palmaria palmata* and *F. vesiculosus* showed the lowest inhibitory concentration in the free radical scavenging antioxidant assays. *Ulva rigida*, *C. tomentosum*, *P. palmata* and *P. dioica* inhibited the cyclooxygenase-2 activity by up to 80%, while *P. dioica* and *P. palmata* extracts showed the highest cytotoxic potential in the MDA-MB-231 breast cancer cells. Altogether, these results support the use of macroalgae as functional foods and promising ingredients for sustainable and healthy diets, in a species-specific context.

The research paper by Al Monla et al. [4] focused on the mechanisms of the cytotoxic and apoptotic effects of extracts from the brown alga *Colpomenia sinuosa* collected on the Lebanese coast. The dichloromethane:methanol extract was the more potent against HCT-116 colon cancer cell line proliferation by increasing the subG1 population of cells and inducing apoptosis. Apoptosis was induced via upregulation of p21 protein and downregulation of the antiapoptotic Bcl 2. This extract also decreased the migration potential of HCT-116 cells with minimal effects on nontumorigenic cells. An increased level of reactive oxygen species was observed in cells treated by the extract of *C. sinuosa* and its effects were inhibited by the addition of an antioxidant. These results indicate that *C. sinuosa* is a source of bioactive compounds possessing proapoptotic and antimigratory efficacy, but the nature of these compounds remain to be elucidated.

Two review papers emphasize the importance of polysaccharides from seaweeds [5,6]. The paper by Ismail et al. [5] explores the chemodiversity of the red seaweeds and underlines their pharmaceutical applications, as well as the exploitation of their specific compounds and secondary metabolites. Rhodophyta are an important group of macroalgae that include approximately 7000 species. They are a rich source of structurally diverse bioactive constituents, including protein, sulfated polysaccharides, pigments, polyunsaturated fatty acids, vitamins, minerals, and phenolic compounds. Polysaccharides are the main components of the cell wall of red algae, of which they account for about 40–50% of the dry weight. They are widely used in the food and pharmaceutical industries because of their thickening and gelling properties. Galactans, carrageenans and agars are the main hydrocolloids of red algae cell wall and have broad-spectrum therapeutic characteristics. The authors also point out that the chemical contents of seaweeds are different according to the algal species, growth stage, environment, and external conditions. Finally, red algae could be economically relevant as a substitute source for natural ingredients that contribute to a wide range of bioactivities such as cancer treatment, anti-inflammatory agents and acetylcholinesterase inhibitors.

Furthermore, the review by Hentati et al. [6] focuses on macroalgae as a source of biomass for the industrial production of bioactive molecules and especially polysaccharides. Indeed, the increasing industrial demand for new biobased molecules requires the availability of efficient and safe biomasses for the identification and production of bioactive metabolites and technofunctional biomolecules suitable for further use in the cosmetic, food and pharmaceutical fields. Among the various biomasses available, macroalgae are gaining in popularity due to their potential benefits in nutraceuticals and health. Several types of molecules are responsible for these health effects such as specific diterpenes, pigments (fucoxanthin, phycocyanin and carotenoids), bioactive peptides and polysaccharides. The review highlights the valuable biological activities of native algae polysaccharides, but also of their derivatives, including oligosaccharides and (bio)chemically modified polysaccharides. However, only a few of them can be developed industrially and open new markets for active molecules, extracts or ingredients. The authors also summarize the health and nutraceutical insight of marine algal polysaccharides and bring an interesting and comprehensive discussion.

The microalgal biodiversity was less explored in the present Special Issue but their variety of potential was exemplified by four papers [7,8,9,10] showing the health application of bioactive molecules from microalgae and cyanobacteria or their use for bioengineering.

This includes a general review of the health benefits of the various molecules that can be extracted from eukaryotic microalgae and cyanobacteria [7]. Moreover, this review by Ferrazzano et al. further emphasizes on oral health, which is very original and opens a new field of application, hitherto little studied, for bioactive molecules from microalgae and cyanobacteria. Indeed, it is well known that due to their richness in high value products, microalgae offer good anti-inflammatory, antioxidant, antitumor, antiglycemic, cholesterol-lowering and antimicrobial activity, but microalgae could also have a significant impact on oral health: several studies agree on the potential application of microalgae for the prevention of oral cancer as well as for the treatment of chronic periodontitis and various oral diseases of microbial origin. Thus, the beneficial effects of microalgae could be implemented in various medical fields. Microalgae and cyanobacteria could represent a potential natural alternative to antibiotic, antiviral or antimycotic therapies, as well as a good complement for the prevention and coadjuvant treatment of various oral diseases. Nevertheless, further studies will be needed to identify the strains of interest, improve overall function and make safe and effective products available to the general population.

The research paper by Mayer et al. [8] provide new insights in the health potential of the well-known microalgae *Phaeodactylum tricornutum*. *P. tricornutum* is a marine microalga, which is rich in bioactive molecules known to be hepatoprotective, such as n-3 long-chain polyunsaturated fatty acids and the carotenoid pigment fucoxanthin. However, its effect on NAFLD, which is a common liver disease characterized by an excess of lipid accumulation, has been poorly studied. Here, the authors have used the hepatocarcinoma cell line HepG2 treated with palmitate as a cellular model of NAFLD. The addition of carotenoid extract (6 μg/mL) or total lipophilic extract (100 μg/mL) prevented the accumulation of triglycerides, total cholesterol and cholesterol esters. Both extracts decreased the mRNA levels of genes involved in lipogenesis (ACACA, FASN, SCD and DGAT1) and cholesterol esterification (ACAT1/SOAT1). By contrast, while the total lipophilic extract also downregulated the LXR/NR1H3 and SREBF1 genes, which are involved in lipogenesis regulation, the carotenoid extract increased the mRNA level of CPT1A, a β-oxidation related gene, and reduced the lipid droplet accumulation. Altogether, the presented results suggest that both *P. tricornutum* extracts may have preventive effects against NAFLD.

The health benefit of microalgae as functional food was also highlighted through the use of *Nannochloropsis gaditana* for nutritional enrichment of farmed salmon [9]. Lozano-Muñoz et al. pointed out that supplementation of commercial diet for Atlantic salmon with 10% of spray-dried *N. Gaditana* significantly increased Vitamin D3, eicosapentaenoic and docosapentaenoic fatty acids levels in boneless and skinless salmon meat. Thus, this original work showed that *N. Gaditana* may be a novel, functional and natural ingredient and a sustainable source of n-3 long-chain polyunsaturated fatty acids that can raise the levels of healthy fats and vitamin D3 in farmed salmon meat.

Finally, Rosales-Mendoza et al. [10] provide an interesting and timely synthesis on the use of microalgae for the production of antiviral compounds and biopharmaceuticals. In the current SARS-CoV-2 pandemic environment, the emergence and continued spread of Coronavirus 2019 (COVID-19) requires the urgent development of antiviral drugs, antibodies and vaccines for prophylaxis. In this review article, antiviral agents with possible activity against SARS-CoV-2 are summarized, based on their previously reported activity against coronaviruses or other enveloped or respiratory viruses. The potential use of algal-derived anti-inflammatory compounds to treat severe cases of COVID-19 is considered. More importantly, the authors highlight and develop the biotechnological use of microalgae to produce vaccines and antibodies. The production of biopharmaceuticals in recombinant microalgae is presented and cases of vaccines made from algae and targeting viral diseases are highlighted as valuable references for the development of SARS-CoV-2 vaccines. In addition, the successful production of functional antibodies in microalgae is described and how specific algal species and genetic engineering techniques can be applied for the production of anti-SARS-CoV-2 antibodies and vaccines are discussed in perspective.

Overall, this special issue will have shown the diversity and the very rich potential of algae and microalgae in functional foods, nutraceuticals, and preventive drugs and as a biotechnology resource for the production of biopharmaceuticals. Although limited to a few specific examples, this panorama augurs well for many developments and has prompted us to renew this theme in a future special issue “Algae and Microalgae and Their Bioactive Molecules for Human Health 2021”.

As guest editor, I would like to thank all the authors for their important contributions, as well as all the reviewers, who greatly contributed to ensuring the high scientific quality of the content. I would also like to thank the authors of the contributions that were not selected and I hope that they will find their place in the next issue. Special thanks to the *Molecules* editorial team, and in particular its managing editor Alvina Wu, for their kind and professional assistance in making this special issue a success. Finally, I hope that this special issue will bring satisfaction and knowledge to the readers.
